# TTV Load Mirrors Local Immunity and Tracks HPV Positivity in the Anogenital Tract

**DOI:** 10.1002/jmv.70850

**Published:** 2026-02-17

**Authors:** Lilia Cinti, Innocenza Palaia, Alessandra Pierangeli, Gabriella D'ettorre, Eugenio Nelson Cavallari, Guido Antonelli, Piergiorgio Roberto

**Affiliations:** ^1^ Laboratory of Microbiology and Virology, Department of Molecular Medicine Sapienza University of Rome Rome Latium Italy; ^2^ PhD of National Interest in Innovation in the diagnosis, prevention and treatment of infections at epidemic‐pandemic risk, Department of Medical Biotechnologies University of Siena Siena Tuscany Italy; ^3^ University Hospital “Policlinico Umberto I” Rome Italy; ^4^ Department of Gynecological, Obstetrical and Urological Sciences Sapienza University of Rome Rome Latium Italy; ^5^ Department of Public Health and Infectious Diseases Sapienza University of Rome Rome Latium Italy; ^6^ PhD National Program in One Health approaches to infectious diseases and life science research, Department of Public Health, Experimental and Forensic Medicine University of Pavia Pavia Lombardy Italy

**Keywords:** Co‐infection, genotype co‐occurrence, human papillomavirus (HPV), mucosal immunity, Torque teno virus (TTV), Viral load

## Abstract

*Torquetenovirus* load has been proposed as an immunomodulated biomarker of host immune status, yet its behavior *in situ* within HPV‐infected mucosa remains poorly defined. We conducted a retrospective cross‐sectional study of 220 patients undergoing HPV screening (181 cervical swabs, 39 anal brushings). HPV was genotyped with Allplex™ HPV28, and TTV load was quantified by in‐house RT‐qPCR and expressed as log_10_ copies per 20 ng total DNA. Analyses included nonparametric group comparisons (Mann‐Whitney) and site‐stratified logistic regression to estimate the TTV‐HPV association. Genotype co‐occurrence was summarized by heatmaps and network analysis and formally tested with Fisher's exact test with Benjamini‐Hochberg FDR correction. TTV load was higher in HPV‐positive subjects (*p* < 0.01), with a significant difference in BR and a trend in CS. In a binary logistic model, each 1‐log_10_ increase in TTV was associated with a 60% increase in the odds of HPV positivity (OR = 1.60, 95% CI 1.07–2.39; *p* = 0.022), with consistent results across CS and BR. TTV load increased with greater genotypic complexity in co‐infections (genotype richness) and was also higher in infections sustained exclusively by HR genotypes than by LR genotypes (*p* < 0.01). The co‐occurrence map and network analysis highlighted recurrent genotype combinations (e.g., 16/53, 53/68, 42/53) and central nodes (16, 31, 51, 56, 68, 53). *In situ* quantification of TTV is associated with HPV positivity and with genotype complexity/risk class, offering local predictive power. The co‐occurrence evidence remains exploratory but supports TTV as an indirect indicator of mucosal immunocompetence.

## 1 Introduction

*Human papillomaviruses* (HPVs) constitute one of the most prevalent viral agents affecting humankind, infecting the epithelial linings of mucosal and cutaneous tissues. Over 200 genotypes have been described to date, of which approximately 40 target the anogenital tract [1]. Among these, a defined subset termed high‐risk (HR) genotypes [2, 3, 4] exerts a well‐established oncogenic potential, representing the necessary cause of cervical squamous cells carcinoma and contributing to the pathogenesis of squamous neoplasms in the anal, vulvar, and oropharyngeal districts. Conversely, low‐risk (LR) genotypes are predominantly associated with benign proliferative lesions, such as condylomas [5]. The most recent IARC classification lists HPV 16, 18, 31, 56, 58 and 66 as Group 1 carcinogens; HPV 68 as Group 2 A (probably carcinogenic to humans); and several other types, including HPV 53 as Group 2B (possibly carcinogenic to humans). Low‐risk types such as HPV 6 and HPV 11 are not classifiable as carcinogenic to humans [4].

Beyond the individual pathogenic roles, HPV infections frequently occur as co‐infections involving multiple genotypes, a phenomenon increasingly recognized as biologically meaningful rather than coincidental [6]. Such viral interplay may shape the local immune microenvironment, modulate viral persistence, and ultimately influence disease outcomes.

In parallel, interest has grown around viral biomarkers capable of mirroring host immune competence. Among these, *Torquetenovirus* (TTV), a small non‐enveloped single‐stranded DNA virus ubiquitously present in humans, has emerged as a promising dynamic indicator of immune status across various clinical contexts, including solid organs transplant recipients, subjects with other kinds of immunosuppression, and individuals with chronic infections [7, 8, 9]. However, the *in situ* TTV behavior and clinical significance within mucosal tissue directly affected by HPV infection, remains largely unexplored.

Given this background, the present study aimed to investigate whether local TTV viral load correlates with HPV infection and genotype representation, exploring potential immune‐virological interactions that may underlie the coexistence and persistence of these viruses within the same anatomical niche.

## 2 Materials and Methods

### 2.1 Study Group

This retrospective study included individuals attending the outpatient clinics of University Hospital Policlinico Umberto I who underwent specimens collection for the detection of HPV infection through molecular methods at the cervical or anal site. Patients with underlying medical conditions or concomitant bacterial or viral infections were excluded to minimize potential confounding factors that could affect the interpretation of the results.

The study was approved by the Local Ethics Committee of Sapienza University of Rome (CE protocol number: 6338), and informed consent was obtained from all participants.

### 2.2 HPV Detection and Genotyping

HPV DNA extraction from CS or BR samples was performed using the STARMag 96 ×4 Universal Cartridge Kit (Seegene, Seoul, Republic of Korea) on the semi‐automated Seegene NIMBUS platform. Detection and genotyping of HPV infection were also carried out on the same platform using the commercial Seegene Allplex™ HPV 28 Detection assay (Seegene, Seoul, Republic of Korea) a qualitative multiplex real‐time PCR assay.

This assay enables the simultaneous amplification and detection of 28 HPV genotypes, including 19 HR‐HPV genotypes (16, 18, 26, 31, 33, 35, 39, 45, 51, 52, 53, 56, 58, 59, 66, 68, 69, 73, 82) and 9 LR‐HPV genotypes (6, 11, 40, 42, 43, 44, 54, 61, 70). The assay relies on molecular discrimination through specific primers and probes, with automated detection of the amplified products. The Allplex™ HPV28 assay is clinically validated and aligned with WHO guidelines, ensuring reliable detection and genotyping across diverse HPV lineages.

### 2.3 TTV Amplification and DNA Normalization

TTV genome amplification was performed using previously extracted genetic material, as described above, which was stored at −80°C until analysis. Total DNA quantification was carried out using the Qubit™ 1X dsDNA HS Assay Kit (Thermo Fisher Scientific, Waltham, MA, USA) and a Qubit™ Fluorometer, which enables the detection of DNA concentrations ranging from 0.2 ng to 100 ng.

To ensure accuracy and comparability of results across samples, DNA quantity was normalized. Specifically, total DNA from each sample was adjusted to a concentration of 2 ng/μL prior to subsequent molecular analyses. This approach minimized variability arising from differences in cellular load or sample quality.

Quantitative detection of TTV was performed using an in‐house real‐time PCR assay, optimized for the detection of all known TTV genotypes, employing a specific set of primers and thermal cycling conditions as previously described by Roberto et al., 2023. To assess potential associations with HPV infection, only samples positive for TTV DNA were included in the analysis. TTV load in these positive samples was then compared according to HPV detection status in both CS and BR specimens. TTV‐negative samples were not included.

### 2.4 Statistical Analysis

Data management, analysis, and visualization were performed in R (v4.3.x) within RStudio (v2025.09.1 + 401). Continuous variables (log_10_‐transformed TTV load, normalized to 2 ng DNA) were compared between two groups using the Wilcoxon‐Mann‐Whitney test (two‐sided). The association between genotype richness (Ngen) and TTV load was quantified by linear regression (effect expressed as mean change in log_10_ TTV per additional HPV genotype; Wald 95% CIs). The relationship between TTV load and HPV detection was evaluated by binary logistic regression, with TTV load as the main predictor; odds ratios were reported per 1‐log_10_ increase and models were run overall and by sampling site (BR, CS).

Co‐occurrence of HPV genotypes was quantified from a patient–genotype incidence matrix (binary: 1 = presence, 0 = absence). Pairwise enrichment of HPV 53 with other genotypes was tested using Fisher's exact test (one‐sided, “greater”); where appropriate (all expected counts ≥ 5), chi‐square with Yates correction was used as a sensitivity check. To account for multiple testing, *p*‐values from pairwise tests were adjusted by the Benjamini–Hochberg false discovery rate (FDR), with significance set at *q* < 0.05. Heatmaps were generated from the co‐occurrence matrix; hierarchical clustering used a binary (Jaccard) distance and complete linkage. Genotype co‐occurrence networks were built with edges weighted by co‐detection frequency and visualized with a force‐directed layout; node degree was used as a centrality descriptor.

Unless otherwise specified, tests were two‐sided with α = 0.05. No imputation was performed; analyses were conducted on complete cases.

In figures, significance is denoted as *p* < 0.05 (*), *p* < 0.01 (**), and *p* < 0.001 (***).

## 3 Results

A total of 220 patients were enrolled; the median age of participants was 42 years (interquartile range [IQR]: 32–54), with a predominance of females (88%). The collected samples included 181 cervical swabs (CS) and 39 anal brushings (BR).

Among all analyzed samples, 67% (*n* = 147) tested positive for HPV DNA: 22% (*n* = 32) were positive for 1 h‐HPV, 13% (*n* = 19) for one low‐risk HPV genotype (LR‐HPV), and the remaining 65% (*n* = 96) showed the presence of two or more HPV genotypes. Specifically, 26% (*n* = 25) of the coinfections involved two or more HR‐HPV, 5% (*n* = 5) involved two or more LR‐HPV, and 69% (*n* = 66) showed the presence of both HR‐HPV and LR‐HPV.

**Figure 1:**
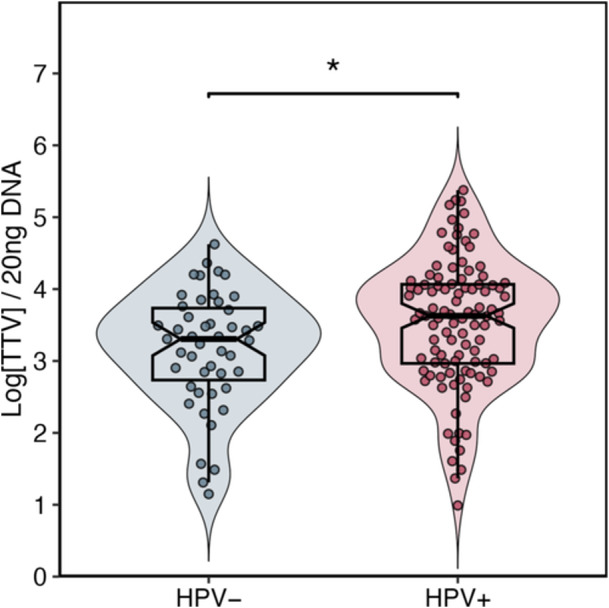
Distribution of TTV load in HPV negative (blue) and HPV positive (red) patients, displayed as a violin plot overlaid with a box plot. Values are reported as log copies per 20 ng of DNA. Boxes indicate median and IQR; points individual observations. TTV load was higher in HPV+ than HPV samples (Mann‐Whitney, *p* < 0.01).

TTV was detected in 68% of the 220 analyzed study samples. In the initial analysis, TTV viral load was compared between subjects who tested positive or negative for HPV. As shown in Figure 1, HPV‐positive participants exhibited significantly higher TTV viral load compared to HPV‐negative subjects (*p* < 0.01).

To better contextualize the observed differences, samples were subsequently stratified as CS or BR. This separate analysis revealed that the difference in TTV viral load between HPV‐positive and negative subjects was statistically significant only in BR, while no significant difference was observed in CS, where only a trend was detected.

**Figure 2:**
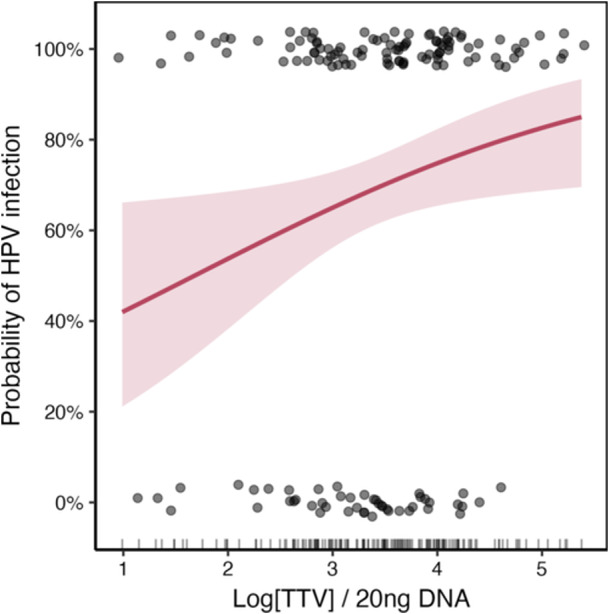
Logistic model of HPV positivity as a function of TTV load. The curve shows the predicted probability with 95% CI (shaded band); points indicate observations (0 = HPV−; 1 = HPV+). Each 1‐log_10 _increase in TTV is associated with 60% higher odds of HPV positivity (OR = 1.60; 95% CI: 1.07–2.39; *p* = 0.022). TTV values are reported as log copies per 20 ng DNA.

To explore the potential predictive role of TTV viral load in relation to HPV infection, a binary logistic regression multivariate analysis was performed. In this model, TTV load was considered the main independent variable and showed a statistically significant association: for each 1‐log_10 _increase in TTV viral load, the odds of being HPV‐positive increased by 60% (OR = 1.60; 95% CI: 1.07–2.39; *p* = 0.022, Figure 2). This effect was confirmed for both CS and BR.

**Figure 3:**
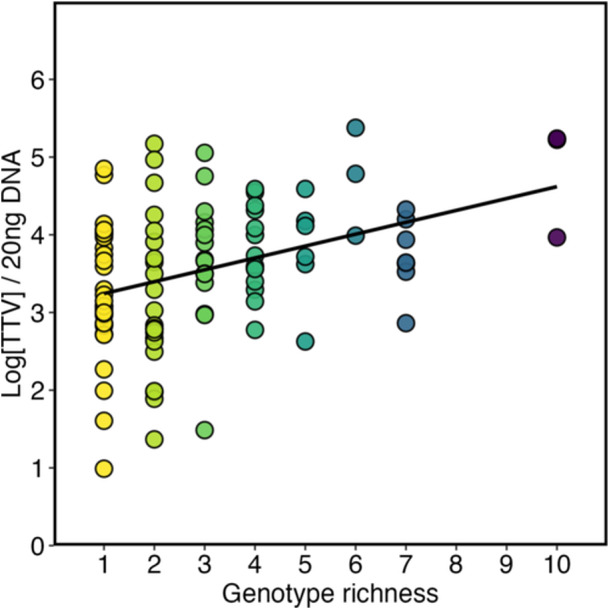
Association between TTV load and HPV genotype richness (number of genotypes per patient). Points represent individual observations; the line shows the regression fit with 95% CI. Each additional genotype was associated with +0.16 log_10 _TTV (*p* = 0.0001). TTV values are reported as log copies per 20 ng DNA.

A further analysis investigated the relationship between TTV load and the presence of a concomitant infections with multiple HPV genotypes. To this end, a composite variable named *genotype richness* (Ngen) was defined, corresponding to the total number of viral genotypes identified in each patient. The results of this analysis showed a positive association between HPV genotype richness and TTV viral load (*p* < 0.01). More specifically, each additional HPV genotype was associated with a 0.16 log units mean increase in TTV viral load (*p* = 0.0001, Figure 3).

**Figure 4:**
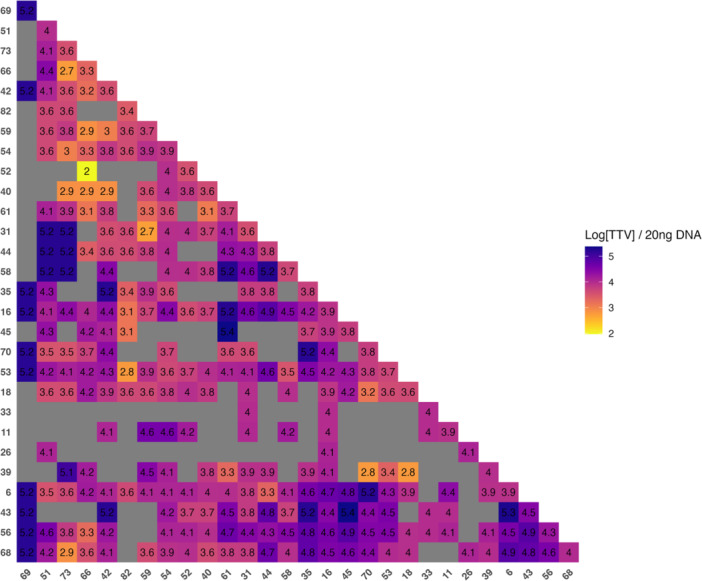
HPV genotype co‐occurrence matrix. Heatmap of pairwise co‐detection frequencies across HPV genotypes; rows/columns are hierarchically clustered to highlight recurrent patterns. Color scale from yellow (lower) to purple hues (higher) indicating increasing co‐occurrence.

Given the observed association between the increase in TTV viral load and local infection due to multiple HPV genotypes, we then analyzed the prevalence of HPV genotypes and the most frequent co‐infection patterns among our study population. The most prevalent genotypes in our cohort, in decreasing order of frequency, were HPV 53, 42, 16, 54, 56, 68, and 6. The co‐occurrence matrix (Figure 4) showed that the most recurrent combinations were HPV 16/53 (*N* = 9), HPV 53/68 (*N* = 8), and HPV 42/53 (*N* = 8). Pairwise Fisher's exact tests suggested higher‐than‐expected co‐occurrence of HPV 53 with HPV 70, 18, 16, and 68 (nominal *p* = 0.016, 0.040, 0.050, and 0.057, respectively; Supplementary Table S1). However, none of these associations remained significant after multiple‐testing correction using the Benjamini‐Hochberg false discovery rate (FDR) (all *q* > 0.05).

**Figure 5:**
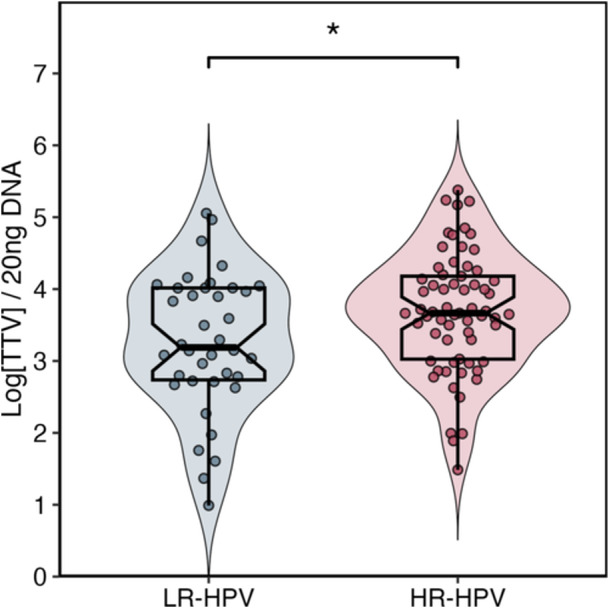
TTV load by oncogenic risk class of HPV genotypes. Violin plot with box plot and individual points for LR‐HPV (low‐risk only) and HR‐HPV (high‐risk only) infections. Values are reported as log copies per 20 ng DNA. TTV load is higher in HR‐HPV than in LR‐HPV infections (Mann‐Whitney, *p* < 0.01).

To assess the potential impact of the oncogenic risk associated with HPV genotypes on TTV viral load, participants were divided into two groups: individuals harboring exclusively HR‐HPV genotypes and subjects with exclusively LR‐HPV genotypes. As shown in Figure 5, participants with HR‐HPV exhibited significantly higher mean TTV loads compared to those with only LR‐HPV (*p* < 0.01).

**Figure 6:**
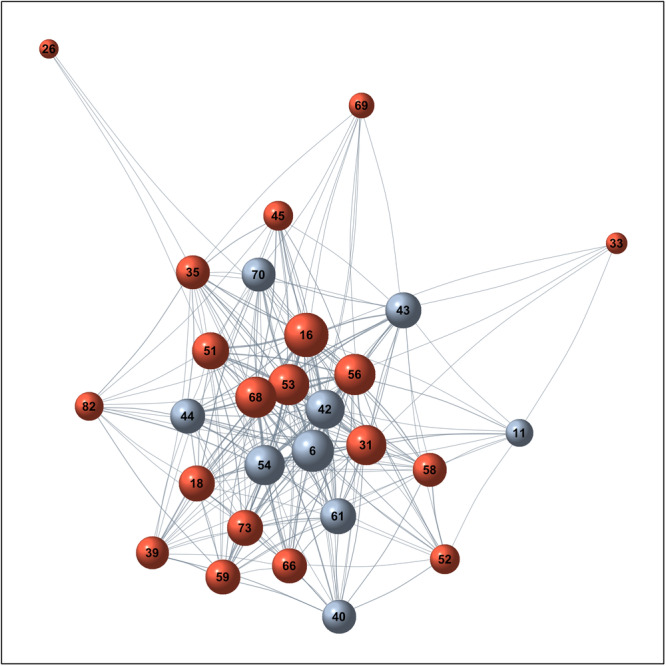
HPV genotype co‐occurrence network. Nodes represent genotypes; edges connect pairs co‐detected in the same patient, with width proportional to co‐occurrence frequency. Colors: red = HR, blue/gray = LR. The graph is displayed with a force‐directed (Fruchterman‐Reingold) layout for visualization only; node positions have no statistical meaning.

Figure 6 shows the co‐occurrence network of HPV genotypes observed among our study population.

This representation revealed that certain genotypes tend preferentially to associate with each other, forming highly interconnected central clusters. Notably, both HR‐HPV genotypes (HPV 16, 53, 56, 68) and LR‐HPV genotypes (HPV 6, 42, 54) are located at central nodes of the network, reflecting frequent co‐detections.

**Figure 7:**
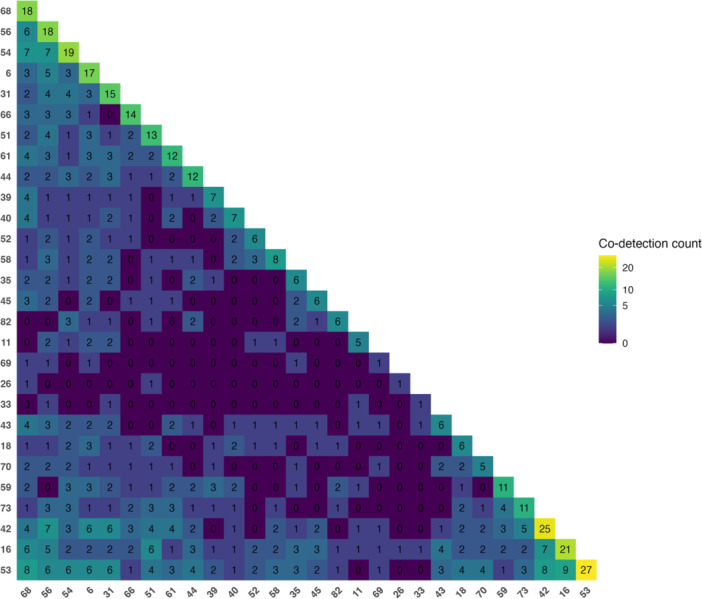
Mean TTV load across HPV co‐infection pairs. Heatmap of mean TTV (log copies per 20 ng DNA) for each HPV genotype pair. Rows and columns denote genotypes and are ordered by hierarchical clustering; cell labels show the mean. Brighter hues indicate higher values; gray cells mark pairs not observed (or with insufficient n).

Finally, to explore the potential impact of genotypes distribution on the extent of TTV viral load, a heatmap was generated to visualize the logarithmic means of TTV load associated with each co‐infection combination (Figure 7). Combinations involving high‐risk oncogenic genotypes, in particular HPV 16, 31, 53, 56, and 68 were associated with higher TTV viral load, represented by brighter yellow/green tones on the map.

## 4 Discussion

In the present study, we investigated the relationship between HPV infection and TTV load in a cohort of 220 patients. Our approach compared samples from two distinct anatomical sites, anal (BR) and cervical swabs (CS). TTV load was evaluated directly from swab collected cells, rather than from blood samples, allowing a more accurate assessment of local interactions between the two viruses and the surrounding host‐tissue microenvironment.

Our results showed that TTV load was higher in HPV‐positive individuals than in HPV‐negative ones. The association was stronger at the anal site, while in cervical samples we observed a similar, although not significant, trend. Nonetheless, applying robust statistical procedures, we identified for the first time a consistent association between increase in TTV load and higher probability of HPV infection in both anatomical sites.

Another key finding was the positive association between TTV viral load and the likelihood of multiple HPV genotypes infection: each additional genotype correlated with a stepwise increase in TTV levels. Moreover, in our population, infections sustained exclusively by HR‐HPV genotypes showed higher TTV load than those involving only LR‐HPV. This finding is consistent with the higher inhibitory effect of HR‐HPV on the local epithelial immune response compared with LR‐HPVs [10, 11, 12, 13], suggesting that the genotypic specificity of HPV infections may shape local TTV abundance, as a reflection of the local immune viral‐host interaction.

To explore HPV community structure, we applied broad genotyping to construct a co‐occurrence heatmap, revealing clustering with recurrent combinations (e.g., HPV 16/53, 53/68, and 42/53), consistent with prior reports [14, 15]. Hierarchical clustering grouped genotypes with shared co‐occurrence profiles, while network analysis identified central hubs (HPV 16, 56, 68, 53) that appear to lead the co‐infection landscape. When associated with TTV load, these patterns suggested that coinfections including HR‐HPV genotypes, particularly HPV 16 and HPV 53, were often associated with higher TTV levels (Figure 7). This supports the hypothesis in which co‐infections involving HR‐HPV genotypes can promote impaired immune surveillance, ultimately favoring TTV replication. Network analysis corroborated these findings, highlighting interconnected core dominated by recurrent genotypes, in particular HPV 53. While this does not imply causality, it raises the surmise that HPV 53 may contribute to co‐infection dynamics, immune evasion, and the stability of viral communities despite its lower intrinsic oncogenic potential, consistent with recent epidemiological data identifying HPV 53 as a frequent participant in co‐infection clusters [16].

Taken together our data indicate that increasing TTV load is associated with a significantly higher probability of observing HPV infection. Furthermore, higher TTV loads were associated with a greater number of HPV genotypes per patient. The increase in TTV load among HPV‐positive subjects, particularly in infections driven by HR‐HPV, supports the hypothesis that TTV may serve as an indirect indicator of local immune status, implying that the *in situ* co‐presence of TTV and HPV may not be purely stochastic. Within this broader framework, HPV, through the activity of its oncoproteins, may not only sustain its own persistence but also facilitate concomitant TTV replication, thereby shaping a permissive microenvironment characterized by attenuated immune surveillance and increased susceptibility to co‐infections. Causality, however, remains unresolved. The co‐presence and replication of TTV and HPV could equally reflect host‐related determinants, such as inter‐individual variability in mucosal immunity, rather than a direct virological interaction.

Additionally, factors related to the time elapsed since HPV infection may influence the current state of the local immune response in mucosal cells, representing an inherent limitation of all cross‐sectional HPV studies. For this reason, it is not possible to evaluate whether TTV load is associated with HPV persistence or clearance over time. Indeed, the natural course of HPV infection, particularly the dynamics of its persistence and eventual clearance in patients, presents significant methodological challenges for study and is, consequently, seldom addressed in the existing literature. Nonetheless, future longitudinal studies integrating virological data with clinical parameters, such as the presence and severity of epithelial dysplasia, would be valuable to elucidate the potential interplay between TTV replication, mucosal immunity, and HPV outcomes.

In conclusion, by relating TTV load to the probability of HPV infection and, in parallel, to the genotype‐level network architecture, our study offers a dual perspective, an integrated conceptual framework for tissue‐level HPV‐TTV interactions and opens new avenues to investigate how viral ecology and the local immune response shape infection outcomes.

## Author Contributions

**Lilia Cinti:** investigation, data curation, formal data analysis, writing – original draft. **Innocenza Palaia, Gabriella D'ettorre:** data resources and clinical investigation. **Alessandra Pierangeli:** visualization, revision, funding acquisition. **Eugenio Nelson Cavallari:** data resources, clinical investigation, revision. **Guido Antonelli:** editing and revision, visualization, supervision. funding acquisition. **Piergiorgio Roberto:** conceptualization, investigation, writing – original draft, statistical analysis, formal data analysis, data curation, funding acquisition. All authors approved the final manuscript.

## Ethics Statement

The study was approved by the Local Ethics Committee of Sapienza University of Rome (CE protocol number: 6338), and informed consent was obtained from all participants.

## Conflicts of Interest

The authors declare no conflicts of interest.

## Supporting information


**Table S1:** Statistical assessment of HPV 53 co‐occurrence with other HPV genotypes.

## Data Availability

The data that support the findings of this study are available on request from the corresponding author. The data are not publicly available due to privacy or ethical restrictions.
