# Supplementary material for: TTV Load Mirrors Local Immunity and Tracks HPV Positivity in the Anogenital Tract
Source: J Med Virol. 2026 Feb 17;98(2):e70850. doi: 10.1002/jmv.70850 (PMC12911470; doi:10.1002/jmv.70850)
Supplement: Supplementary file 1 — Table S1: Statistical assessment of HPV 53 co‐occurrence with other HPV genotypes. [file JMV-98-e70850-s001.docx]

**Table S1.** Statistical assessment of HPV 53 co-occurrence with other HPV genotypes

| **HPV genotype pair** | **Observed count** | **OR** | ***p-*value** | **FDR** |
| --- | --- | --- | --- | --- |
| 53-70 | 4 | 12.483 | 0.017 | 0.387 |
| 53-18 | 4 | 6.206 | 0.041 | 0.387 |
| 53-16 | 9 | 2.597 | 0.054 | 0.387 |
| 53-68 | 8 | 2.706 | 0.057 | 0.387 |
| 53-31 | 6 | 2.078 | 0.165 | 0.723 |
| 53-35 | 3 | 2.962 | 0.187 | 0.723 |
| 53-43 | 3 | 2.962 | 0.187 | 0.723 |
| 53-69 | 1 | Inf | 0.265 | 0.763 |
| 53-6 | 6 | 1.653 | 0.267 | 0.763 |
| 53-42 | 8 | 1.431 | 0.317 | 0.763 |

HPV, *Human papillomavirus*; OR, Odds ratio; FDR, False discovery rate
